# Treatment of Sleep Disordered Breathing Liberates Obese Hypoxemic Patients from Oxygen

**DOI:** 10.1371/journal.pone.0140135

**Published:** 2015-10-09

**Authors:** Marcus Povitz, Patrick J. Hanly, Sachin R. Pendharkar, Matthew T. James, Willis H. Tsai

**Affiliations:** 1 Department of Community Health Sciences, University of Calgary, Calgary, Alberta, Canada; 2 Department of Medicine, Western University, London, Ontario, Canada; 3 Department of Medicine, University of Calgary, Calgary, Alberta, Canada; 4 Sleep Centre, Foothills Medical Centre, Calgary, Alberta, Canada; Charité—Universitätsmedizin Berlin, GERMANY

## Abstract

**Background:**

Obese hypoxemic patients have a high prevalence of sleep disordered breathing (SDB). It is unclear to what extent treatment of SDB can improve daytime hypoxemia.

**Methods:**

We performed a retrospective cohort study of obese hypoxemic individuals, all of whom underwent polysomnography, arterial blood gas analysis, and subsequent initiation of positive airway pressure (PAP) therapy for SDB. Patients were followed for one year for change in partial pressure of arterial oxygen and the need for supplemental oxygen.

**Results:**

One hundred and seventeen patients were treated with nocturnal PAP and had follow-up available. Adherence to PAP was satisfactory in 60%, and was associated with a significant improvement in daytime hypoxemia and hypercapnea; 56% of these patients were able to discontinue supplemental oxygen. Adherence to PAP therapy and the baseline severity of OSA predicted improvement in hypoxemia, but only adherence to PAP therapy predicted liberation from supplemental oxygen.

**Conclusions:**

The identification and treatment of SDB in obese hypoxemic patients improves daytime hypoxemia. It is important to identify SDB in these patients, since supplemental oxygen can frequently be discontinued following treatment with PAP therapy.

## Introduction

The prevalence of obesity has increased in the last decade to over 25% of the population [1, 2]. We have previously reported that the predominant cause of hypoxemia in this population is sleep disordered breathing (SDB) rather than chronic obstructive pulmonary disease (COPD) [3]. Treatment of such individuals with oxygen alone may be harmful by worsening SDB [4–6] and missing an opportunity to implement appropriate therapy with continuous positive airway pressure (CPAP) or non-invasive ventilation (NIV). These therapies have been associated with improved health outcomes [7–10].

Sleep disordered breathing is highly prevalent among obese hypoxemic individuals; with obstructive sleep apnea (OSA) found in 80%, the obesity hypoventilation syndrome (OHS) in 51%, and sleep hypoventilation in 83% [3]. Previous studies have shown that CPAP or NIV therapy may improve hypercapnea and hypoxemia in patients with hypercapneic OSA [11] or COPD [7]. However, it is unclear from these studies whether the magnitude of response was sufficient to discontinue supplemental oxygen, or how this compared to improvement in hypoxemia in patients managed without CPAP or NIV.

The primary objective of this study was to determine the proportion of obese hypoxemic patients who were able to discontinue supplemental oxygen following initiation of positive airway pressure therapy (PAP). The secondary objective was to identify predictors of improvement in gas exchange.

## Methods

### Study design

A retrospective cohort study was performed in all obese (body mass index (BMI)>30 Kg/m^2^), and hypoxemic (arterial partial pressure of oxygen (PaO_2_) <60 mmHg) patients referred for assessment with both arterial blood gas (ABG) and polysomnography (PSG) between January 1 2009 and October 30th 2013. All PSG testing was performed at the Foothills Medical Centre (FMC) Sleep Centre. The FMC Sleep Centre is the only publicly funded adult sleep laboratory in Calgary, Alberta (population ~1.1 million). Polysomnography was required during this time to satisfy the Alberta government funding criteria for home oxygen therapy for obese individuals. Additionally, all patients found to have SDB, were required to undergo treatment with PAP. Hypoxemia was confirmed by ABG analysis performed during the PSG setup while the patient was upright and awake. Patients were followed for adherence to PAP therapy and improvement in gas exchange for a minimum of 1 year. This time period was chosen to allow patients ample opportunity to optimize their adherence to PAP therapy and to benefit from it.

### Exposure

The exposure of interest was adherence with either CPAP or NIV, also referred to as PAP therapy. We included three groups of obese hypoxemic patients: those not-treated for SDB, those treated for SDB who were adherent, and those treated for SDB who were not adherent. The choice of PAP was at the discretion of the treating physician. Adherence was defined as either: patients having (a) PAP download showing 4 hours of use on 70% of nights in the first 6 months following PSG (adherent group), or (b) clinical note of satisfactory adherence with therapy by an experienced clinician (clinical adherence group). All patients with SDB were offered follow up at the FMC Sleep Centre to improve treatment adherence. The FMC Sleep Centre comprises physicians who are accredited in respiratory and sleep medicine, and respiratory therapists with extensive expertise in SDB therapies. Ventilatory equipment (CPAP, oxygen concentrators, NIV) was provided by private home care companies under contract with the Alberta government. Setup and maintenance of the equipment was the responsibility of the home care company but was verified by the FMC Sleep Centre staff.

### Outcome

The outcomes of interest were: (a) improvement in daytime PaO_2_ at follow up in the first year compared to baseline, and (b) the percentage of patients able to discontinue daytime oxygen in the first year of follow-up. A patient was able to discontinue oxygen if they had an oxygen saturation >90% or a PaO_2_ > 60 mmHg while breathing room air. These are the criteria for prescription of supplemental oxygen therapy in Alberta and are therefore clinically relevant for patients and the health care system [12]. Cessation of oxygen is a meaningful outcome given possible harm associated with its use in patients with OHS[4–6]; and its lack of efficacy in comparison to CPAP for treating OSA[13, 14]. Monitoring of arterial blood gasses or oxygen saturation were a requirement for continued oxygen funding. The alveolar-arterial gradient was calculated using the formula (Atmospheric pressure-47)*0.21-arterial partial pressure of carbon dioxide (PaCO_2_)/0.8-PaO_2_; atmospheric pressure in Calgary is 670mmHg.

### Covariates

The presence of SDB was determined from a baseline PSG performed in all patients. The PSG provided continuous monitoring of electroencephalogram (3 channels: C3, C4, O1) electrooculogram (2 channels, left and right), electromyogram (sub-mental), single lead electrocardiogram, oro-nasal thermistor, nasal airflow, abdominal and thoracic respiratory effort, pulse oximetry, and transcutaneous partial pressure of carbon dioxide. Data were collected using Sandman software [Sandman v8.0 NATUS Embla Systems, Tonawanda, NY, USA]. PSG studies were scored using the Canadian Thoracic Society criteria [15]. An obstructive apnea was defined as a ≥90% reduction in airflow for at least 10 seconds with persistent respiratory effort. A central apnea was defined as a ≥90% reduction in airflow for at least 10 seconds without respiratory effort. A hypopnea was defined as a ≥50% reduction in airflow lasting at least 10 seconds or any reduction in airflow for at least 10 seconds accompanied by a >3% drop in oxygen saturation or an arousal.

Obstructive sleep apnea was defined as an apnea hypopnea index (AHI) > 5. Mild OSA was defined as an AHI of >5 to <15, moderate OSA ≥15 to <30, and severe OSA as ≥30. Central sleep apnea was defined as an AHI >5 where more than 50% of the respiratory events were central. The obesity hypoventilation syndrome was defined as PaCO_2_ during wakefulness ≥45mmHg in the presence of obesity (BMI≥30 kg/m^2^) and absence of other causes of hypoventilation such as chronic lung disease [16]. Sleep hypoventilation was defined as an increase in transcutaneous PCO_2_ ≥ 10mmHg on transition from wakefulness to sleep or an absolute level > 55mmHg during sleep [17].

Co-morbidities were determined by chart review, and enhanced [18] from hospital discharge abstracts to determine comorbidities contained in the Charlson index [19]. The diagnosis of COPD was determined by spirometry using the Global initiative for chronic Obstructive Lung disease (GOLD) criteria. COPD was defined by a forced expiratory volume in 1 second (FEV1) to forced vital capacity (FVC) ratio of < 0.7. Severe COPD was defined by an FEV1 < 50% of predicted.

### Statistics

Baseline characteristics were reported using means and standard deviations, and proportions with 95% confidence intervals. Differences between groups were tested using ANOVA for normally distributed characteristics, the Kruskal-Wallis test for non-normally distributed characteristics and the Pearson’s chi-square test for proportions. A p-value of <0.05 was considered statistically significant for all testing. The percentage of patients in each group able to discontinue oxygen was expressed as a proportion with 95% confidence intervals. The associations between PAP treatment adherence and liberation from oxygen were determined using the Pearson’s chi square test, stratified according to treatment type (CPAP and NIV). Change in blood gas values from baseline to follow up was tested using paired t-tests. Linear regression was performed to assess associations between change in oxygenation and age, sex, BMI, baseline A-a gradient, FEV1, FVC, FEV1/FVC<0.7, AHI, presence of sleep hypoventilation, common comorbidities and adherence with PAP therapy. Interactions between PAP therapy adherence and sleep hypoventilation, an FEV1/FVC <0.7 and the A-a gradient were tested but found to be non-significant, and were thus not included in the final multivariable model. Given that the non-respiratory comorbidities were not significantly associated with change in oxygenation (p>0.05) in univariate analysis they were also excluded from the final multivariable model to avoid overfitting. Logistic regression was performed to identify predictors of discontinuation of supplemental oxygen therapy during follow-up. These included age, sex, BMI, AHI≥30, FEV1/FVC<0.7, FEV1<50%, A-a gradient, common comorbidities and adherence with PAP therapy as independent variables. All were first tested separately and then together in a full multivariable model. Interactions between PAP therapy adherence and sleep hypoventilation, an FEV1/FVC <0.7 and the A-a gradient were considered but not included in the final models as they were found to be statistically non-significant. Again the non-respiratory comorbidities were excluded to avoid overfitting. All analyses were performed with STATA IC 13 (Stata Corp, Texas USA).

### Ethics statement

This study was approved by the conjoint research ethics board of the University of Calgary (REB13-0032).

## Results

### Patients

**Fig 1:**
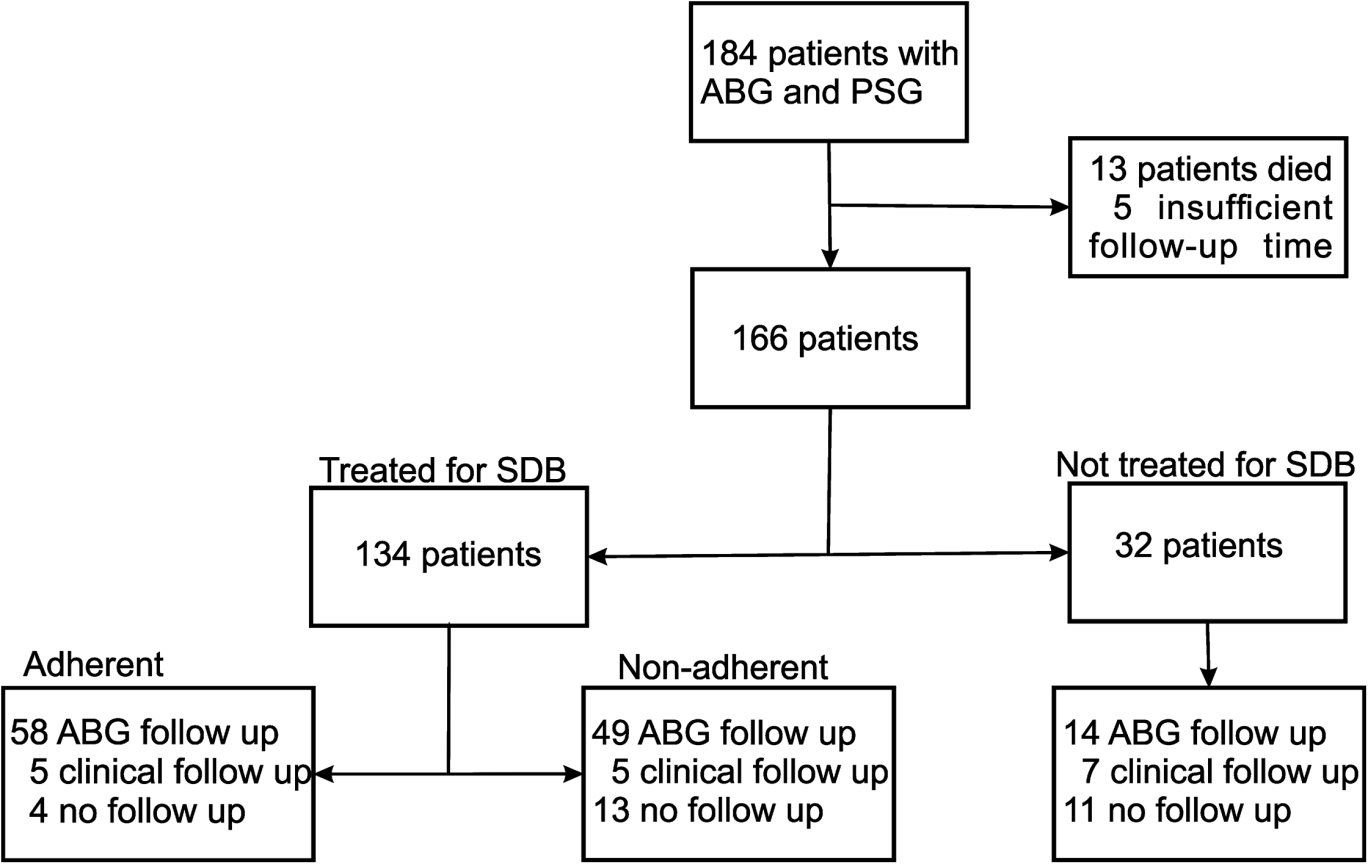
Study flow diagram. ABG arterial blood gas, PSG polysomnograph.

One hundred and eighty four patients had a baseline PSG and ABG assessment (Fig 1). Insufficient follow-up time was available for 18 individuals, 13 of whom died before one year had elapsed. Follow up ABG’s were available for 121 individuals and 17 additional individuals had documented oxygen saturations, breathing room air, at approximately one year of follow-up. Of the 28 individuals missing follow up ABG’s, 11 were not treated for SDB. Of the 138 individuals with follow up measurements of oxygenation, 117 were treated for SDB. The two groups of adherent patients were quite similar (Table 1). Patients were treated with CPAP (n = 46) or NIV (N = 87). One patient who was treated with adaptive servo-ventilation was excluded.

**Table 1:** Baseline characteristics of all patients with follow-up (N = 138).

	Not treated (n = 21)	Clinical adherence (n = 35)	Adherent (n = 28)	Non-adherent (n = 54)	P value
Age* years	61.6 (16.8)	58.2 (10.9)	62.8 (14.4)	59.7 (11.5)	0.508
Sex male/female	10/11	20/18	18/10	25/29	0.409
BMI* Kilogram/metre^2^	39.2 (8.7)	45.2 (8.3)	44.8 (8.6)	44.1 (8.4)	0.055
**Arterial blood gas**					
	N = 21	N = 35	N = 28	N = 54	
Baseline pH*	7.42 (0.03)	7.42 (0.03)	7.41 (0.04)	7.41 (0.03)	0.243
Baseline PaO_2_ * mmHg	52.5 (5.7)	50.5 (7.1)	51.4 (4.8)	49.8 (5.9)	0.301
Baseline PaCO_2_ * mmHg	43.9 (5.6)	49.9 (8.8)	48.6 (7.2)	47.4 (6.4)	0.022
Baseline A-a gradient*, mmHg	23.4 (7.1)	17.9 (10.8)	18.7 (8.5)	21.8 (8.5)	0.062
**Spirometry**					
	N = 18	N = 31	N = 25	N = 46	
FEV1*, percent predicted	60.8 (20.1)	63.1 (19.3)	62.7 (18.9)	64.0 (19.7)	0.950
FVC*, percent predicted	72.5 (15.3)	71.1 (16.7)	73.2 (18.7)	72.5 (18.2)	0.974
FEV1/FVC*, percent	65.5 (17.0)	71.3 (13.1)	67.5 (8.1)	70.9 (11.8)	0.296
**Sleep disordered breathing**					
	N = 21	N = 35	N = 28	N = 54	
AHI*, events/hour	18.3 (32.0)	76.2 (56.5)	69.0 (48.0)	49.0 (45.9)	<0.001
OSA severity(none/mild/mod/severe)	8/7/4/2	4/3/4/24	2/2/4/20	6/11/10/27	0.001
OHS^$^, percent	29 (8–50)	63 (46–80)	57 (38–77)	56 (42–69)	0.081
**Prevalence of comorbidities,**					
	N = 21	N = 35	N = 28	N = 54	
Hypertension^$^	62 (39–85)	80 (66–94)	68 (49–86)	65 (52–78)	0.404
COPD^$^	56 (30–81)	35 (18–53)	60 (39–81)	48 (33–63)	0.642
Severe COPD^$^	22 (1–43)	16 (2–30)	20 (3–37)	26 (13–39)	0.958
Congestive heart failure^$^	43 (20–66)	34 (18–51)	32 (14–51)	44 (31–58)	0.641
Diabetes Mellitus^$^	38 (15–61)	40 (23–57)	25 (8–42)	44 (31–58)	0.392
Kidney disease^$^	14 (0–31)	9 (0–18)	7 (0–17)	9 (1–17)	0.853
Malignancy^$^	5 (0–15)	11 (0–23)	7 (0–17)	6 (0–12)	0.721
Myocardial infarction^$^	10 (0–23)	11 (0–23)	0 (0–0)	0 (0–0)	0.026

*Mean (standard deviation)

^$^ Proportion (95% confidence interval)

Abbreviations: A-a gradient alveolar to arterial gradient, AHI apnea hypopnea index, BMI body mass index, COPD chronic obstructive pulmonary disease, FEV1 forced expiratory volume in 1 second, FVC forced vital capacity, OSA obstructive sleep apnea, OHS the obesity hypoventilation syndrome, PaO_2_ arterial partial pressure of oxygen, PaCO_2_ arterial partial pressure of carbon dioxide, mmHg millimeter of mercury, 95%CI 95% confidence interval

### Need for supplemental oxygen

**Fig 2:**
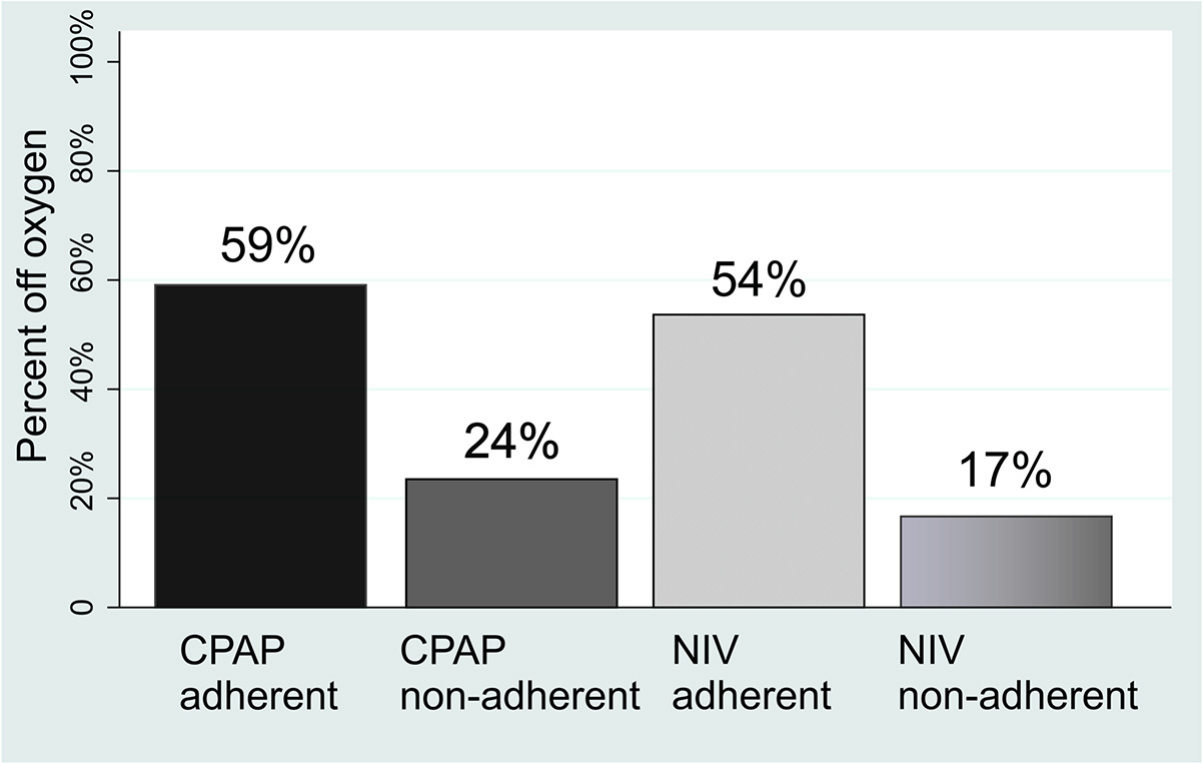
Improvement in need for supplemental oxygen therapy in patients treated with PAP therapy. CPAP continuous positive airway pressure, NIV non-invasive ventilation.

Significant improvements in oxygenation were seen during the 12 months of follow-up without a change in the A-a gradient. Larger improvements were seen for the adherent groups although there was some improvement for non-adherent groups as well (Table 2). Adherence with CPAP and with NIV was 56% (95% CI 40%–72%) and 63% (95% CI 52%–74%) of patients respectively. There was no significant difference in gas exchange improvements associated with CPAP and NIV, so these groups were combined for analysis of oxygen discontinuation. Oxygen was discontinued in 39% (95%CI 30%, 48%) of all patients who were started on PAP and this increased to 56% (95%CI 43%, 68%) in those who were adherent with PAP therapy. The proportion of adherent patients who could discontinue oxygen was similar for those who were started on CPAP (59%) and NIV (54%) (p = 0.6950) (Fig 2). In contrast, supplemental oxygen could only be discontinued in 20% (95%CI 9%–31%) of non-adherent patients. The relative proportion of patients able to discontinue supplemental oxygen was higher for patients adherent with CPAP than those who were non-adherent (Relative risk 1.86, 95%CI 1.06–3.30, p = 0.0264), and for patients adherent with NIV than those who were non-adherent (RR 2.03 95%CI 1.36–3.03,) p = 0.008). The A-a gradient did not change significantly in spite of therapy.

**Table 2:** Initial and follow-up blood gas values according to therapy and adherence.

	PaO_2_, mmHg			PaCO_2_, mmHg			A-a gradient, mmHg		
CPAP	Baseline	Follow-up	p-value	Baseline	Follow-up	p-value	Baseline	Follow-up	p-value
Adherent (n = 8)	52.4 (3.6)	60.8 (7.1)	0.0260	46.1 (4.6)	42.5 (6.3)	0.1200	20.8 (7.1)	17.0 (8.5)	0.1158
Clinically adherent (n = 12)	50.8 (5.4)	59.9 (8.7)	0.0213	46.0 (6.6)	40.0 (4.7)	0.0209	22.5 (6.6)	21.0 (9.2)	0.4077
Not adherent (n = 16)	52.1 (4.0)	54.3 (5.4)	0.1678	46.3 (5.9)	44.3 (5.0)	0.1214	20.8 (7.0)	21.2 (6.9)	0.8462
NIV	Baseline	Follow-up	p-value	Baseline	Follow-up	p-value	Baseline	Follow-up	p-value
Adherent (n = 16)	51.1 (5.8)	60.3 (7.9)	0.0027	50.9 (7.6)	44.0 (4.9)	0.0047	16.2 (8.2)	15.5 (9.1)	0.8136
Clinically adherent (n = 22)	49.9 (7.9)	59.9 11.6)	0.0004	52.3 (9.3)	44.7 (7.0)	0.0026	15.5 (12.1)	15.1 (11.7)	0.8452
Not adherent (n = 32)	48.8 (6.3)	53.0 (8.6)	0.0192	48.1 (7.1)	45.8 (5.6)	0.0436	21.9 (8.3)	20.6 (10.0)	0.4667

Abbreviations: A-a gradient alveolar-arterial gradient, PaO_2_ arterial partial pressure of oxygen, PaCO_2_ arterial partial pressure of carbon dioxide, mmHg millimeter of mercury, CPAP continuous positive airway pressure, NIV non-invasive ventilation, mmHg millimeters of mercury

The patients not treated for SDB were less obese, had milder gas exchange abnormalities and tended to have less SDB and worse pulmonary function. In the subgroup where follow up was available 57% (95%CI 34%, 80%) no longer required supplemental oxygen (p = 0.1238).

### Improvement in Hypoxemia

The apnea hypopnea index, and adherence with PAP therapy were associated with improvement in PaO_2_ (Table 3). Baseline A-a gradient, spirometric values or sleep hypoventilation were not associated with significant improvements. In the final multivariable linear regression model, adherence was associated with an improvement in PaO2 as was higher baseline AHI. However, male gender was significantly associated with worsening PaO_2_ during follow up.

**Table 3:** Predictors of improvement in PaO_2_.

Univariable		Difference, mmHg(95%CI)
	Age, per year	0.0 (-0.1–0.1)
	Sex, for males	-1.5 (-5.1–2.1)
	BMI, per kilogram/meter^2^	0.1 (-0.1–0.3)
	Baseline A-a gradient, per mmHg	0.02 (-0.23–0.18)
	AHI, per event/hour	0.05 (0.02–0.08)*
	Sleep hypoventilation	1.2 (-4.2–6.5)
	FEV1, per percent predicted	0.1 (0.0–0.2)
	FVC, per percent predicted	0.0 (-0.1–0.1)
	FEV1/FVC <70	-2.8 (-6.4–0.81)
	Adherence with PAP	5.7 (2.1–9.4) *
	Hypertension	3.1(-0.73–7.0)
	Congestive heart failure	1.0 (-2.6–4.7)
	Diabetes	-1.5 (-5.2–2.3)
	Chronic kidney disease	-1.4 (-7.6–4.9)
	Malignancy	1.3 (-5.2–7.8)
Multivariable		
	Sex, for males	-4.7 (-8.6 –-0.71) *
	AHI, per event/hour	0.08 (0.04–0.12) *
	Adherence	6.0 (2.1–9.8) *

*Statistically significant

Abbreviations: A-a gradient alveolar arterial gradient, AHI apnea hypopnea index, BMI body mass index, FEV1 forced expiratory volume in 1 second, FVC forced vital capacity, 95%CI 95% confidence interval.

Adherence to CPAP or NIV was the only univariable predictor of a patient not requiring supplemental oxygen after one year of follow-up (OR: 4.89 (2.13, 11.18) (Table 4). Adherence to PAP therapy also remained significantly associated with becoming independent of supplemental oxygen following multivariable adjustment for age, sex, BMI, and respiratory parameters (OR:6.6 (2.52–17.24)).

**Table 4:** Predictors of discontinuing supplemental oxygen.

Univariable		Odds ratio (95% CI)
	Age, per year	0.99 (0.96–1.02)
	Sex, for males	0.92 (0.47–1.81)
	BMI, per kilogram/meter^2^	0.99 (0.95–1.03)
	AHI≥30	1.04 (0.53–2.04)
	Sleep hypoventilation	0.45 (0.16–1.29)
	Baseline PaCO_2_≥45mmHg	0.66 (0.32–1.35)
	A-a gradient	0.98 (0.95–1.02)
	FEV1<50% predicted	0.81 (0.35–1.87)
	FEV1/FVC<70%	0.66 (0.33–1.32)
	Hypertension	1.34 (0.64–2.80)
	Congestive heart failure	0.81 (0.40–1.62)
	Diabetes	0.58 (0.28–1.18)
	Chronic kidney disease	1.21 (0.38–3.79)
	Malignancy	0.91 (0.25–3.40)
	Adherence	4.89 (2.13–11.18) *
Multivariable		
	Adherence	6.6 (2.52–17.24)*

*Statistically significant

Abbreviations: A-a gradient alveolar arterial gradient, AHI apnea hypopnea index, PaCO_2_ arterial carbon dioxide pressure, BMI body mass index, FEV1 forced expiratory volume in 1 second, FVC forced vital capacity, 95%CI 95% confidence interval

## Discussion

Our study shows that adherence to CPAP or NIV was associated with an improvement in gas exchange in obese hypoxemic individuals. Over half of patients who were adherent to PAP therapy could discontinue supplemental oxygen within one year of starting PAP. This occurred without a significant change in the A-a gradient; suggesting that treatment of hypoventilation was responsible for the improvement. Regression analysis showed that the improvement in gas exchange was associated with AHI and adherence with PAP therapy, but not with baseline A-a gradient or sleep hypoventilation_._ Adherence with PAP therapy was the only predictor of becoming independent of supplemental oxygen in both univariable and multivariable analysis.

The finding that improvement in PaO_2_ was not associated with baseline A-a gradient is surprising, since it would have been expected that those with hypoventilation would benefit most from PAP therapy. Consequently, without a trial of therapy it is impossible to predict if oxygenation might improve. The lower improvement in PaO_2_ associated with male gender is likely multifactorial. Previous research suggests that central obesity, found more commonly in males, [20] is more strongly associated with hypoxemia[21]. In addition, males may have been heavier smokers [22], which was not measured.

To our knowledge, this is the first study to examine the association between treatment of SDB and improvement in daytime oxygenation in unselected obese hypoxemic individuals. Previous studies have reported on cohorts of patients with OHS or with COPD. The studies of OHS patients have not reported a comparison between treated and untreated individuals. These include studies by Budweiser et al [23] and Perez et al[24] who reported results only for patients adherent with PAP therapy. Priou et al reported an overall improvement in oxygenation; however patients who were non-adherent with PAP therapy were not analysed separately as a control group[6]. In contrast, our study compared outcomes in three groups of patients, namely those who did not receive treatment with PAP, those who did receive PAP and were not adherent and those who received PAP and were adherent. Additionally, since polysomnography was a requirement for oxygen funding in obese hypoxemic patients, the potential for selection bias was reduced.

Several randomised control trials of NIV treatment for COPD have recently been reported. Struik et al randomised patients recovering from acute respiratory failure to NIV or usual care and found no difference in improvement in hypoxemia, or mortality [25]. Kohnlein et al reported that in COPD patients with stable chronic respiratory failure [26], NIV improved mortality but did not improve gas exchange significantly compared to a control group similar to the earlier study by McEvoy et al [27]. In contrast, we report an improvement in oxygenation associated with treatment. This may be attributable to our cohort being obese as well as having significant SDB.

The group of patients in our study who were not prescribed PAP therapy had significantly less severe gas exchange abnormalities, a lower prevalence of SDB and less severe OSA as reflected by a lower mean AHI. The group BMI was also lower for the untreated group, although this was not statistically significant. These differences may explain the improvement in hypoxemia among patients not treated with CPAP or NIV. This may also represent a survivor bias as fewer untreated patients had ABG follow up. The lesser degree of improvement noted in the non-adherent group may represent benefit from delayed or partial adherence with PAP or improvements related to treatment of other comorbidities.

This study has some limitations, firstly, we could not locate follow up ABG results in some patients; however, the majority of these patients were also non-adherent with PAP therapy. It is likely these results would have increased the size of the benefit seen with PAP therapy, since the non-adherent patients in the treated arm experienced less spontaneous improvement in PaO2 compared to those who did not receive PAP therapy. Secondly, medical therapies other than PAP may have contributed to the improvement in gas exchange and have confounded our findings if patients adherent with PAP were more likely to be adherent with these therapies. However, Villar et al have reported that medication non-adherence is not worse in those who are CPAP non-adherent [28]. Thirdly, we did not have detailed information on the severity of co-morbidities, which may have impacted outcome measurements. However, the prevalence of co-morbidities was similar between adherent and non-adherent groups. Fourthly, measurements of BMI were not available at follow-up. Previous research has shown that changes in weight are associated with improvements in hypoxemia [29]. However, significant weight reduction is uncommon in patients with SDB and, furthermore, it is unlikely that patients would have lost weight preferentially in the PAP-adherent group [30]. Finally, we used a criterion of PaO_2_ < 60 mmHg, to indicate independence from supplemental oxygen, rather than a composite of 55 and 60 mmHg, plus pulmonary hypertension or polycythemia. However, since one of our ABG inclusion criterion was a PaO_2_ < 60mmHg, we wanted to use the same value to define the ability to discontinue oxygen, as using a lower value would have artificially increased the rate of oxygen discontinuation.

## Conclusions

In obese hypoxemic patients with SDB, adherence with CPAP or NIV therapy is associated with significant improvement in daytime gas exchange and discontinuation of supplemental oxygen. Investigation and treatment of SDB and optimization of PAP therapy should be part of the conventional management of chronic hypoxemia in obese patients.
